# Rapid Design of a Student-Centred App for Musculoskeletal Clinical Skills: An Example of a Theoretically Informed Approach to Developing Apps for Learning

**DOI:** 10.5334/pme.1223

**Published:** 2024-06-27

**Authors:** Tehmina Gladman, Henry Li, Oliver McCullough, Rebecca Grainger

**Affiliations:** 1 University of Otago Wellington, New Zealand

## Abstract

**Background and need for innovation:**

The process to design mobile apps for learning are infrequently reported and focus more on evaluation than process. This lack of clear process for health professional education mobile apps may explain the lack of quality mobile apps to support medical student learning.

**Goal of innovation:**

The goal of this project was to develop a student informed ready for production wireframe model of a minimally viable mobile app to support learning of musculoskeletal (MSK) clinical skills.

**Steps taken for development and implementation of innovation:**

The Information Systems Research (ISR) framework and Design Thinking were combined for the mobile app design. The process followed the cycles and modes of the combined framework to; systematically review available apps, use a focus group to identify attributes of the app valued by students, define the initial plan for the mobile app, develop an app prototype, and test and refine it with students.

**Outcomes of innovation:**

The student focus group data had five themes: 1) interactive usability, 2) environment, 3) clear and concise layout, 4) anatomy and pathology, 5) cultural safety and ‘red flags’. The prototyping of the app went through three cycles of student review and improvement to produce a final design ready for app development.

**Critical reflection on our process:**

We used a student-centred approach guided by design frameworks to design a minimally viable product mobile app to support learning of MSK clinical skills in ten weeks with a small team. The framework supported nonlinear, iterative, rapid prototyping. Student data converged and diverged with the MSK teaching methods literature. Of note our students requested cultural safety learning in the app design, suggesting mobile apps could support cultural safety learning.

## Background and need for innovation

### Background

While education-related mobile apps proliferate, to date the processes used to design a quality mobile app for medical education are infrequently reported [1]. The studies that describe app development generally focus on practicalities of design and evaluation of the completed app, rarely describing the design process [2]. This lack of a clear design process for health professions education apps may explain the lack of quality mobile apps available to support medical student learning [3].

### User-centred mobile app design frameworks

There are several processes that can inform app design. The AGILE framework describes a process of rapid iteration of prototypes to test multiple designs with users and determine the best design to take to the development phase [4]. The LEAN framework is an example of rapid iteration of ideas to determine likely candidates for development [1]. While these examples make use of consumer feedback to support the iteration process, the Information Systems Research (ISR) framework is specifically a user-centred design with three research cycles: Relevance Cycle – ensuring the app is relevant and useful to its target audience; Rigour Cycle – requiring the app to be evidence based; and Design Cycle – requiring an iterative building process with consistent feedback from end-users and experts [5]. The ISR framework is non-linear and iterative allowing rapid prototyping and feedback on designs [6]. While the ISR framework has shown practical success in application design, researchers have noted weaknesses which may be mitigated by incorporating design thinking [7].

Design thinking is an approach to problem solving that uses the end user in a solution-focussed approach [7]. It consists of five modes: empathise – observation, engagement and immersion in end-user experience; define – analysis and synthesis of empathise mode learning to define the need; ideate – brainstorming possible solutions; prototype – turning solution ideas into testable activities; and test – gathering feedback, learning from users, and refining solutions. Each mode interacts with the others non-linearly and iteratively in the design process [8].

Farao and colleagues noted that while both ISR and design thinking include the end-user in the design process, design thinking centres the end-user in both the process and the solution. ISR comes from design science and has a positivist perspective while design thinking has a more constructivist perspective [9]. Thus, the weakness in ISR is strengthened through the centring of the user voice, and the weakness in design thinking is strengthened through robust scientific methods [7]. In their paper, Farao and colleagues described combining the two processes using a case study of a redesign of a patient health app [7].

### Case study: Musculoskeletal conditions

Musculoskeletal (MSK) conditions are a global health burden, accounting for at least 15% of primary care presentations [10,11]. However, doctors and medical students report lacking knowledge and confidence in MSK assessment [12,13]. Some literature suggests competence in the MSK examination may not be consistently achieved, possibly due to inadequate MSK-focused curriculum in medical programmes [14,15]. We propose that an MSK examination skills app could support learners to gain MSK knowledge in clinical settings, particularly for “just-in-time” learning. However, most previous literature in health app development has focused on apps for educating patients [16,17]. As such, any design of an app to support medical students’ or doctors’ proficiency in MSK assessment will need to be informed by the wider educational literature.

### Methodologies to support MSK learning

A review of the literature shows several learning methods, including hypothesis-driven physical examination (HDPE) [18], near-peer teaching [19], focused anatomy teaching [20], and self-directed learning [21], may be effective in student learning of MSK examination skills and may be appropriate in a mobile app. To understand the current MSK mobile app landscape, apps in the two major app stores were evaluated with the Mobile App Rubric for Learning (MARuL) tool. The MARuL is a validated measure of educational app quality. Apps are scored under four categories: teaching and learning, user centred measures, professionalism, and usability [22]. The review identified four apps that supported MSK learning; pGALS, Musculoskeletal Pro Consult, Physiopedia, and Orthopedic Examination & Special Tests. While each included elements of the methodologies described above, only Physiopedia scored a high enough score on the MARuL [22] to be considered a recommended student learning app; however, it was aimed specifically at physiotherapists. There was no quality MSK app for learning that targeted medical student needs.

## Goal

Our goal was to develop a student informed ready for production wireframe model of a minimally viable mobile app to support learning of musculoskeletal (MSK) clinical skills, informed by Farao et al’s ISR and design thinking [7].

## Steps taken for development

**Figure 1:**
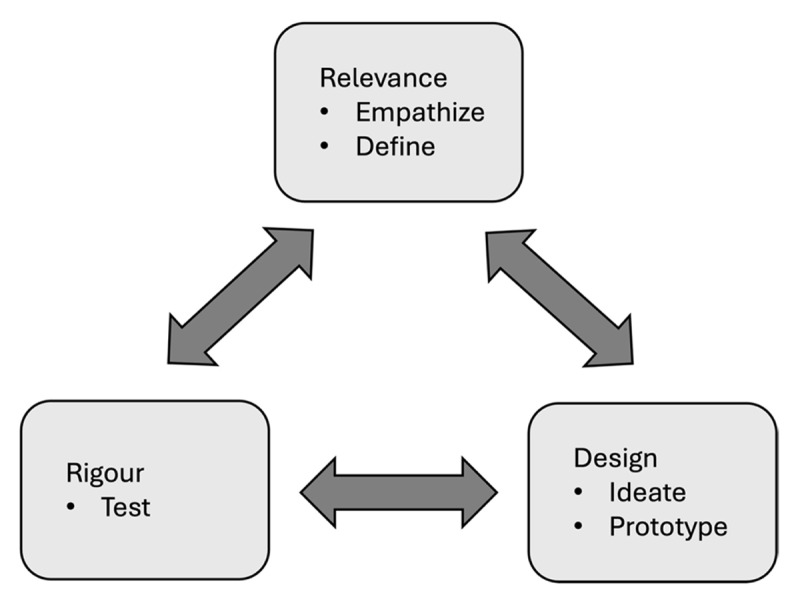
Combining the Information Systems Research (ISR) Framework with Design Thinking [5,7].

The team wanted to design a fit-for-purpose minimally viable product (MVP) mobile app wireframe incorporating student learning needs and functionality requirements for a quality MSK examination skill learning app [7]. A fit-for-purpose MVP is a ready to develop wireframe model incorporating the minimum useful and usable learning activities as described by the literature and our end-users, the students. We followed the steps described by Farao and colleagues incorporating both ISR and design thinking into our process (Figure 1) [7]. Ethics approval was gained from University of Otago Human Ethics Committee Ref: D21/340 and D21/341. Consent for participation was obtained prior to each step of the process as needed.

### Relevance cycle – Empathise mode

To meet the empathise mode goals we used a focus group representing the end-user of a MSK examination skills learning mobile app. We invited Year 5 and 6 medical students at our university, recruited via the snowball method, to a focus group to discuss their ideas of an ideal MSK mobile app [23]. Year 5 and 6 students were chosen due to their focus on clinically-based learning and because they are an anticipated key end-user group for an MSK examination skills mobile app. The ten-question semi-structured interview (Appendix 1) was informed by the literature on effective MSK learning methodologies, combined with the findings from the app review described in the introduction.

### Relevance cycle – Define mode

The focus group data were analysed using general inductive analysis [24], transcribed using otter.ai, and checked for accuracy by one of the authors (OM) [25]. Data were managed using Microsoft Word (2022) due to its ease and familiarity. In the first cycle of coding, data were topic coded using the learning methodologies described in the introduction [26]. Comments which did not fit those methodologies were topic coded separately. Pattern coding was then used in the second cycle to organise the data into themes relating to student need in a MSK clinical skills mobile app for learning [26].

### Design cycle – Ideate mode

In the ideate mode we focussed on the literature, app search results, and opinion from the team; a rheumatologist (RG), educational technologist (TG), and two medical students (OM & HL); to develop the initial app design. Aspects of mobile app usability described in the wider education literature such as aesthetic design, auditory richness, user-friendliness, self-assessment, interactive content and scientific comprehensiveness were considered for the app design [27,28]. For example, ‘gamification’ has been found to increase user retention in educational apps while research has shown that a community section, such as fora, in medical educational apps may be useful in supporting peer-to-peer learning [29,30,31]. Furthermore, endorsement by local universities and associated organisations support better uptake by medical students and junior doctors [32].

### Design cycle – Prototype mode

To design the first draft of the wireframe model, we made decisions regarding the content and aesthetics of the app based on the results of the ideate mode. To make the design more acceptable to student expectations, we designed it based on an iPhone X, a phone commonly used in our student cohort. We used open-source icons from The Noun Project (https://thenounproject.com) with appropriate attribution. For simplicity the colour scheme was largely black and white.

Figma (https://www.figma.com) was used to create a basic outline of the wireframe model, including content and features. Figma is free software offering a prototyping feature that displays the wireframe model on an interactive smartphone screen. The initial wireframe model was reviewed and edited by the team. This review and editing cycle was repeated twice, ending with the first complete draft wireframe prototype.

### Rigour cycle – Test mode

Medical students were invited to participate in a series of “think-out-loud” style interviews to test and provide feedback on the draft wireframe model. “Think-out-loud” requires the participant to speak aloud everything that comes into their mind as they complete an activity. These thoughts allowed the interviewer to accurately understand what parts of the wireframe model are more or less intuitive [33].

The study population for the interviews were year 4–6 medical students from the University of Otago. To recruit participants, convenience and snowball sampling methods were used [23]. The same students were invited to test the wireframe model and be interviewed twice.

The interviews were conducted over Zoom (2022). Participants were sent a link to the wireframe model, asked to screen share and “think-out-loud” as they navigated the wireframe model. After completing the “think-out-loud”, students were asked three follow-up questions: “Was there anything confusing about the app,” “What is important to you in a good MSK examination skills app” and “Would you use this app, and if not what would need to change?” Participants then completed the System Usability Scale (SUS) to measure the useability of the wireframe model [34]. The System Usability Scale is a well-validated 10-item measure to quickly assess the usability of a product. It has been previously used for rapid assessment of mobile app usability for rheumatoid arthritis patients with good results [35]. Each interview was recorded, transcribed and feedback was summarised by HL to determine updates to the model.

After each of the two rounds of interviews, the feedback gained from participants was evaluated with the team and used to guide the development of the next draft of the wireframe model until the model was brought to pre-production quality (final design). During these iterations, results from the Define Mode focus group were included as they became available. There were three iterations of the wireframe model and two rounds of interviews and surveys. We compared the SUS scores from the first round of feedback to those from the second round of feedback.

## Outcomes

In this section we describe the outcome of each stage of our design process, ending with the completed production ready wireframe MSK mobile app. We also briefly describe the costs in time and resources to complete a design process of this type.

### Relevance cycle – Empathise and Define mode

Seven year 5 and 6 medical students (six female, one male) participated in a 55-minute focus group. Student ethnicities included three pākeha (New Zealand European), three Asian and three Pasifika (students could identify as multiple ethnicities).

We identified five key themes: 1) the need for interactive usability, 2) environment, 3) clear and concise layout, 4) anatomy and pathology, 5) cultural safety and ‘red flags’. Students described the need for interactive usability by noting, for example, the usefulness of “a snapshot of a group of muscles, something that you can like spin around and like, visualize dynamic and interactive”. Another student described the ideal “videos on how to do each thing … not watching like a 10-minute video, but like a 30 second video … would be really useful to keep refreshing yourself on how to do it …”.

When discussing the environment, the focus group suggested that the most effective time and place for a MSK examination app would be just-in-time-learning. One student noted that “I think it would have a lot of value like, in those weird settings, … on your own at GP or something, when you’re not specifically thinking about … ortho … and have it in front of you”. While another student thought they would be most likely to use the app “in the stairwell between the paeds [paediatrics] ward and the ED”.

Students felt that a clear and concise layout could be achieved through consistent sections, subsections and return to home functions. They felt this consistency would give them the ability to determine for themselves “if you need to see the whole exam from introduction to differentials and management plan … or you can kind of have subheadings that you can go into”.

The focus group suggested the equal importance of organising app content by anatomy and pathology to cover MSK examination approaches for patient presentations with both normal joints or structures (e.g. anatomy of a normal knee joint) and pathological states of joints or structures (e.g. examination of a knee joint with severe synovitis in the presence of inflammatory arthritis). One student thought it would be useful “If there was like a tag on everything, … conditions would pop up, and you could just go by that tag”.

Students clearly expressed that cultural safety was an important aspect to highlight within a clinical skills mobile app. They thought that this could be best accomplished through reminders or ‘don’t forget’ prompts. For example, one student thought that “Maybe a red flag section because like, we talk a lot … about red flags”, while another student gave the suggestion that “Like if you’re going to examine someone’s head? Yeah. Having a little side note to double check that they’re okay with that”.

### Design cycle – Ideate and prototype mode

#### Initial design of wireframe

**Figure 2:**
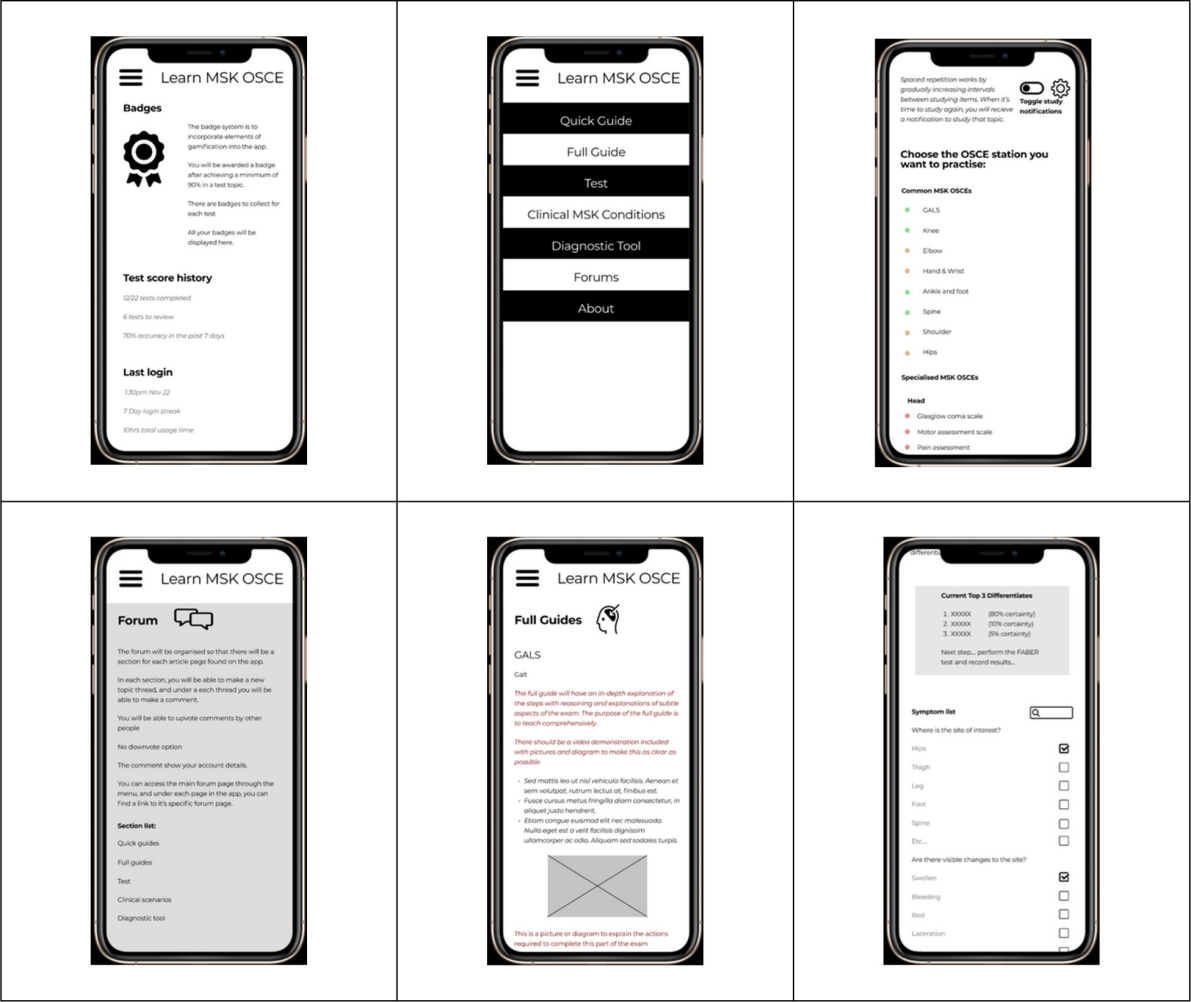
From top left to bottom right: Home page, Menu, OSCE testing, Forums, Full guides & Diagnostic tool.

The initial wireframe design included the following sections; quick guides, full guides, test, clinical conditions, diagnostic tool, badges, forums, and “about” information. The wireframe included mock ups of the intended content/functions (see Appendix 2 for descriptions). Figure 2 shows screenshots of the first draft of the wireframe model.

### Rigour cycle – Test mode

#### Interview rounds

**Figure 3:**
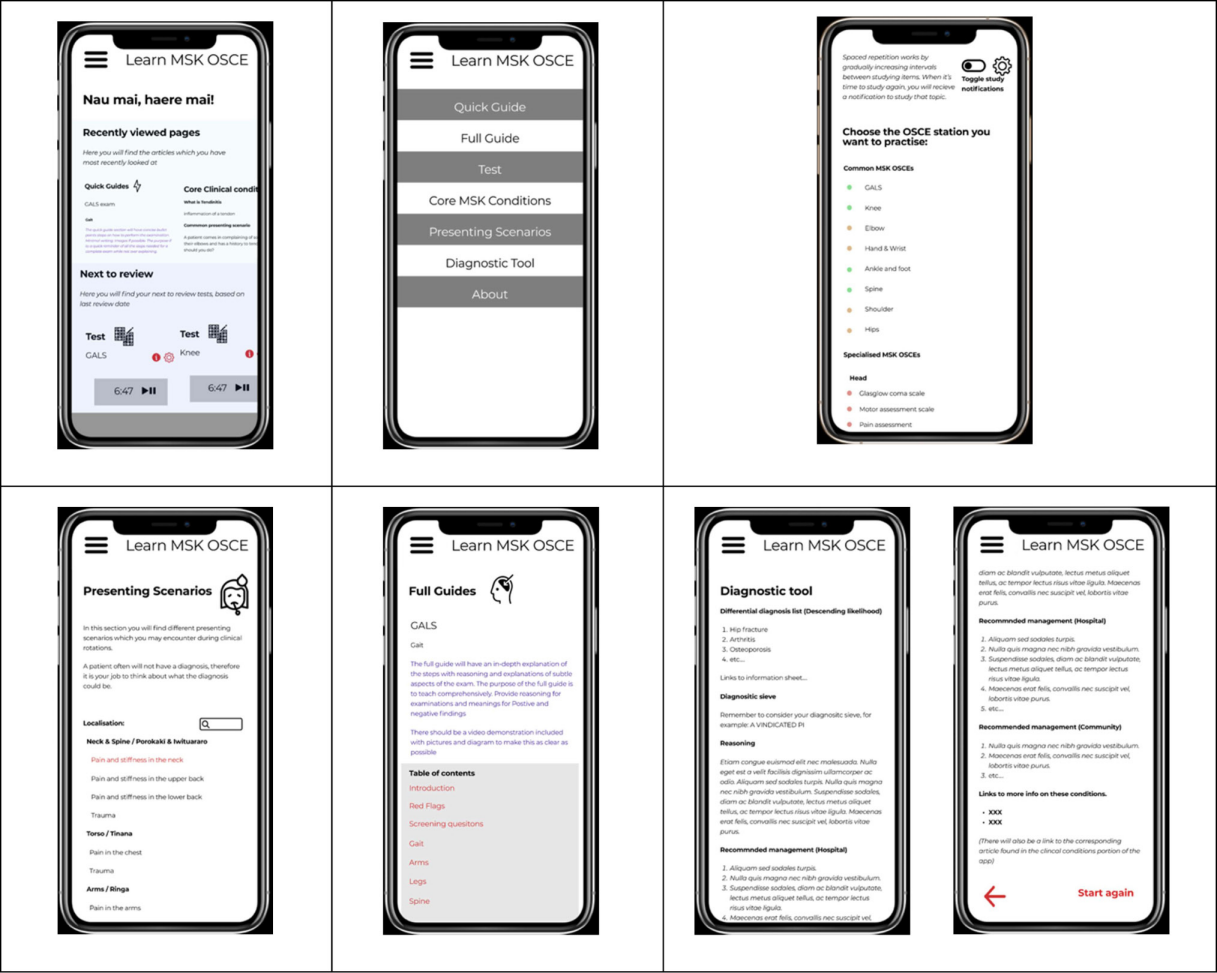
From top left to bottom right: updated home page, updated menu, OSCE testing with badging removed, added presenting scenarios, updated full guides with table of contents, final version of diagnostic tool with diagnostic sieve and location management.

Six students participated in the interviews. For each round, the same six students were interviewed using the think-aloud technique as they tested the wireframe model. The results of each of the three design rounds from initial ideated prototype through to final design can be found in Appendix 2. Figure 3 shows the final version of several of the updated and added features.

#### System Usability Scale (SUS)

The sample size for the System Usability Scale were the six students who completed the Rigour cycle – Test Mode. Using the Sauro-Lewis CGS [34], we interpreted the means for the SUS for Round 1 and Round 2 as an A+ with a Round 1 mean of 90.8 (SD = 6.65) and a Round 2 mean of 92.9 (SD = 5.34). The Wilcoxon signed-rank tests [36], used due to the small sample size, indicated that the score for the second round of the SUS was not significantly higher than the first round, Z = 7.00, p = 0.26. However, a plot of the scores indicated that the variance between scores for the six interviewees decreased by approximately 36% for the second survey (Round 1 Var = 44.2, Round 2 Var = 28.5) suggesting that the second iteration was more consistently rated by the participants.

### Cost of design work

We calculated approximate costs of mobile app design to support student learning using this process. We considered monetary, time, and skills costs. By our estimation, the total monetary costs were NZ$12,000 for two summer students, plus .1 FTE of lecturer and .05 FTE Associate Professor salaries for ten weeks. We also paid for an educational subscription to Otter.ai at a cost of US$79.99 for a one-year subscription. We estimate that the project took ten weeks full time for each of the two summer students, forty hours from a lecturer, and twenty hours from an associate professor. The only special skills our group had were an interest in educational technology and MSK for the students and, for the lecturer and associate professor, previous educational technology experience.

## Critical reflection

Using a combined design framework of ISR and design thinking as described by Farao and colleagues [7] we described key elements of the literature, assessed the current state of available mobile apps for MSK learning, engaged with end-users, brainstormed an initial solution, prototyped the app as a wireframe through iterative feedback from the end-users, and finalised a potential minimally viable product in the span of 10 weeks. While we described a linear process, the relevance and design cycles occurred concurrently, and iterative changes were made as new data were integrated. The combined ISR and design thinking framework allows for this type of concurrent, non-linear iterative design to be undertaken to rapidly develop a design that could be passed to a development team for realisation.

Using our findings from the literature review and the assessment of currently available apps to develop the focus group questions for the relevance cycle allowed us to determine if the student viewpoint converged with that of the literature and the strengths and weaknesses of currently available apps. We found that while students did agree with the literature on several points including the need for anatomy and pathology [20], the need for active learning [21], and the opportunity to focus exams on hypothesized differentials [18]; students did not consider near-peer teaching a necessary aspect of a mobile app. Students also offered additional learning methods that were not found in the literature, such as the need to include cultural and language awareness, important in New Zealand, and the need for just-in-time reminders. This convergence and divergence from the literature highlights the importance of including the end-user experience from the beginning of any design process [6,7].

Concurrent with the relevance cycle, we began developing the design cycle ideate mode. This allowed us to develop potential solutions that could be informed further by data from our end-users, the students. The development of an initial solution set gave us the ability to create an initial mobile app prototype to show students something to critique during the test mode of the rigour cycle. By developing the initial solution concurrently with the empathise mode of the relevance cycle we were also able to speed up the process to the prototype mode of the design cycle.

Of particular interest during these cycles of design and development were students’ requests for the inclusion of reminders to support cultural awareness in the New Zealand context. This is an example of students and staff collaborating as “agents of change” for decolonisation in a curriculum [37] by creating opportunities to support learning of cultural safety, examples of which would be of use to anyone working in a country with indigenous or historically marginalised groups for which a better understanding of culturally safe medical interactions would improve health outcomes.

We did encounter challenges in designing the wireframe model. For example, due to the timing of the project (during the summer break), we chose to use convenience plus snowball sampling for both the focus group and the interviews. As a result we may have missed important voices within the student body. A further challenge for us was the tight timeframe for the design process. This meant that we only spoke with students from one campus of the medical school potentially limiting the relevance of the app beyond that campus. Finally, although our research team composed of academics and students worked well together, we did need to spend significant time ensuring that all members were on the same page throughout the project. While this represented a significant learning experience for the students members, it is important to point out the significant time needed to give more structured research supervision to students at the undergraduate level.

The process we used shows that with a small team it is possible to design a model fit-for-purpose app that keeps the end-user firmly at the centre while including a strong evidence-base and making improvements over currently available apps. By focussing on people rather than technology, an app can be developed that students are likely to use and which can improve their learning.

## Appendix

### Appendix 1. Focus group questions

1. Tell me about any apps you have used to support your learning in medical school
  - Prompts: use of websites, use of apps, why or why not
2. Tell me about any MSK related apps you have used to support your learning in medical school
  - Prompts: use of websites, use of apps, why or why not
3. There are several parts of a MSK examination: screening tests, look-feel-move, applied anatomy and special tests – what type(s) of content would be important to have in an MSK examination app in order to support your learning?
  - Prompts: Overview of a joint examination, bullet pointed/condensed information, anatomy, videos, common presentations
4. Tell me about what would be important in the interface of a MSK examination app
  - Prompts: Illustrations, full text, mixture, real images, videos
5. Tell me about the clinical setting or environment that an MSK examination app would be useful for
  - Prompts: OSCE study, Just in time learning, Orthopaedics, Rheumatology, ED, GP, Gen med
6. Often after we do a physical examination we are asked to formulate a differential diagnosis; tell me what would be useful in a MSK examination app that would support this part of your learning?
7. Tell me what would be important to have in a MSK examination app that would support and encourage cultural safety
8. Much of a MSK examination is reliant on understanding of anatomy; tell me how incorporating anatomy might or might not be useful for a MSK examination app
9. Bedside teaching is a common and useful part of clinical medicine; tell me what would be useful in an MSK examination app that would relate to or be useful for bedside teaching
10. The further you progress through medicine, more self-directed learning is required; tell me how a MSK examination app would support self-directed learning.

### Appendix 2 Design Cycle, ideate and prototype mode

**Table:**

DESIGN ELEMENTS	PROTOTYPE STAGES				

	INITIAL DESIGN	EXAMPLE QUOTES FROM INTERVIEW FEEDBACK ON INITIAL DESIGN	ITERATION 2	EXAMPLE QUOTES FROM INTERVIEW FEEDBACK ON ITERATION 2	FINAL VERSION

Home page	Included	Does the information on the home screen come up every time you open the app? That would be annoying	Updated to include recently viewed pages	The home page is nice with the recently viewed pages and spaced repetition	Included

Quick guide – bullet point notes and diagrams	Included	Having links between the quick guide and full guide would be useful so you can quickly flip back and forth	Updated to include linking between quick and full guides	The links between the quick guide and full guides are nice	Included

Full guide – in-depth descriptions and comprehensive video demonstrations	Included	Organising the full guide page with a table of contents would be better	Updated with table of contents	Table of contents is good	Included

Test – function to test examination skills. In each test, there is a timer and a checklist of all steps in the examination with a ‘push’ reminder function using spaced repetition learning methods. [38]	Included	More detailed summary page to the test section would be good	Included	The spaced repetition feature in the test section is quite cool, I like that	Included

Clinical conditions – brief descriptions of conditions in the curriculum and were intended to support learning about MSK-related conditions	Included	In terms of organizing items in a list, potentially order them alphabetically would be easier	Updated organization of conditions	The links to find more information on topics are nice, because something you want to read up more about it and the links make it faster to access more information	Included

Diagnostic tool – checklist of symptoms, envisaged to help medical students and junior doctors brainstorm differential diagnoses	Included	Include a flowchart instead of ticking the table in the diagnostic tool	Adapted to visual flowchart to increase interactivity	The diagnostic tool now is very intuitive to use and flows very well	Included

Badges –awarded “badge” icons to user profiles as users completed activities in the app	Included	Not quite sure about the badge system here	Removed (feedback suggested that gamification could make the app feel less professional and inappropriate for on the ward learning)	–	–

Forum – opportunity to develop a community of users	Included	Clinical accuracy, breadth of content and ease of use would be the most important criteria	Removed (concern that lack of information regulation could lead to inaccurate information)	–	–

About page – acknowledge relevant parties and provide information on the origin of the app	Included	Would be more likely to use if the app is endorsed and promoted by the university	Included	It’s good that you have references for your information	Included

Search bar	–	A search bar would be handy; Have a search bar as scroll can be annoying	Included	Search bar makes things easier	Included

Red flags*	–	–	Included	In the red flag section, maybe add reminders of important things to check like GCS	Included

Cultural awareness*	–	–	Included	Some reminders about when you touch a Māori patient’s head, ask are good; Make sure that the Māori integration doesn’t become tokenism	Included

Te reo Māori (New Zealand official language) *	–	–	Included	Te Reo integration has been done well	Included

Diagnostic sieve	–	–	–	Maybe add a diagnostic sieve for differential diagnosis in each of the sections so students can be more systematic when they approach thinking of a cause for a presentation	Included

Community vs hospital management	–	–	–	The management for different presenting complaints vary depending on the setting, for example are you in the hospital or are you in the community as a GP?	Included

*Updated based on combined focus group and interview data.
